# Considerations on Visible Light Communication security by applying the Risk Matrix methodology for risk assessment

**DOI:** 10.1371/journal.pone.0188759

**Published:** 2017-11-29

**Authors:** Ignacio Marin-Garcia, Patricia Chavez-Burbano, Victor Guerra, Jose Rabadan, Rafael Perez-Jimenez

**Affiliations:** 1 Telematics Engineering Dept. Facultad de Ingenieria en Electricidad y Computacion, Escuela Superior Politecnica del Litoral (ESPOL), Guayaquil, Ecuador; 2 Instituto para el Desarrollo Tecnológico y la Innovación en Comunicaciones (IDeTIC), Universidad de Las Palmas de Gran Canaria, Las Palmas de Gran Canaria, Las Palmas, Spain; Victoria University, AUSTRALIA

## Abstract

Visible Light Communications (VLC) is a cutting edge technology for data communication that is being considered to be implemented in a wide range of applications such as Inter-vehicle communication or Local Area Network (LAN) communication. As a novel technology, some aspects of the implementation of VLC have not been deeply considered or tested. Among these aspects, security and its implementation may become an obstacle for VLCs broad usage. In this article, we have used the well-known Risk Matrix methodology to determine the relative risk that several common attacks have in a VLC network. Four examples: a War Driving, a Queensland alike Denial of Service, a Preshared Key Cracking, and an Evil Twin attack, illustrate the utilization of the methodology over a VLC implementation. The used attacks also covered the different areas delimited by the attack taxonomy used in this work. By defining and determining which attacks present a greater risk, the results of this work provide a lead into which areas should be invested to increase the safety of VLC networks.

## Introduction

Visible Light Communications (VLC), defined by its Institute of Electrical and Electronics Engineers (IEEE) standard [1], similar but mistakenly known as LiFi [2], is one of the newest communication technologies that may be massively used in the next few years [3]. As a broadband communication technology, VLC is expected to be part of the upcoming heterogeneous networks complementing other technologies such as five generation telephony (5G), WiFi and Ethernet [4, 5]. An axiom in network security is that a network is as secure as its weakest link. Among the highlighted strengths of this technology, security is usually listed at the top. The security attributed to VLC is generally associated with the belief of light being confined by walls. Therefore, VLC data streams cannot be easily observed from outside the rooms or premises where they are generated. However, few security studies have been done regarding the intrinsic security of VLC, and therefore a safety concern comes into our mind when implementing VLC based networks. This concern is based on the expected large amounts of data that, in the near future, will be transmitted through VLC networks [6, 7]. In addition to this, the information transmitted through VLC, as the one used on geolocation techniques [8–12], could be exploited for criminal activities, since the transmitted data could be of great interest for potential attackers. For the stated reasons, further understanding of the security limitations of VLC and its exploitation should be studied and understood for the users protection.

Regarding those reasonable concerns about security, some general research into VLC security has been conducted. Mostafa and Lampe studied the use of null-steering and artificial noise strategies to achieve positive secrecy rates against eavesdropping attacks [13]. In [14], the same authors considered using friendly jamming to secure data transmissions. In [15], Blinowski studied the risk of snooping, jamming and modifying VLC-based communications. In [16], Al-Kinani et al. evaluated the power received in a VLC system using simulations and a novel field-of-view (FOV) geometry-based single bounce (GBSB) model which results pointed out that wireless optical channel was highly correlated at the center of the environment and the correlation decreased gradually when moving towards the environment edges. In [17], Classen et al. considered the theoretical eavesdropping possibility of VLC based communications through keyholes and door gaps. In [18], a lab test of VLC sniffing using components-off-the-shelf (COTS) was performed with positive results. Finally, in [19], Prasad et al. compared Ultra-Wide-Band (UWB) and VLC for data intensive and security sensitive applications. This work concluded that VLC was a good option from a cost and interference point of view and complemented other data transfer technologies yet further work in VLC reliability and privacy was needed. All the presented works began to consider and tried to determine some security and secrecy boundaries for VLC transmissions. With the upcoming release of the revision of the standard [20] by the IEEE 802.15.7r1 Task Group, the opportunity to address security concerns present itself. By analyzing standard attacks and using standardized tools or risk assessment, adequate focus in the more insecure characteristics of VLC can be taken.

Notwithstanding a short use on [15], and a few others examples for qualitative risk assessment, Risk Matrices are not usually found in the literature to evaluate risk in wireless communication. However, this approach is widely used in multiple fields where qualitative evaluation is looked for or even required. For instance, matrices were used in [21] to assess the risk on a campus-wide network. In [22] the risk matrix approach was used in a software project risk management assessment. In [23], this methodology was used for supply chain risk assessment, while in [24], it was used to assess the risk while driving. In [25] this approach was used to evaluate the Information Technologies (IT) outsourcing risk, and in [26] it was applied to measure security risks for smartphones. All these examples are just a few of the large set of literature available works in which, for almost all areas of knowledge, Risk Matrices are used to assess hazards and determine which areas, or attacks, should be prioritized for risk mitigation.

## Constructing the Risk Matrix for VLC systems

A Risk Matrix is a structured approach to the risk assessment process used in project and security management. Risk Matrices allow the identification of the potential impact and the probability of occurrence in a visual way and assist decision making. The method was proposed by the United States Air Force Electronic System Center (USAF-ESC) in 1995, ant it was included in 2000 as part of the Military Standard (MIL-STD) 882D [27]. The objective of this methodology is to determine risk existence. Based on the project needs and technical possibilities, the method gives a qualitative impact level. Finally, this methodology provides a Risk Rank or risk level that allows management to focus the resources on the prevention of potentially disastrous problems rather than in low-level risk events.

**Fig 1:**
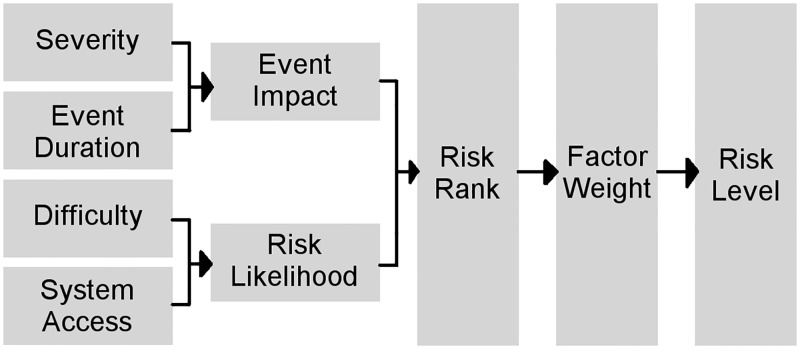
Risk Matrix model. The process of risk modeling starting from four initial inputs (Severity, Event Duration, Difficulty and System Access) to create a Matrix of Experts. The evaluated matrix generates the event’s Impact and the Likelihood values that serve to generate the Risk Rank.

**Fig 2:**
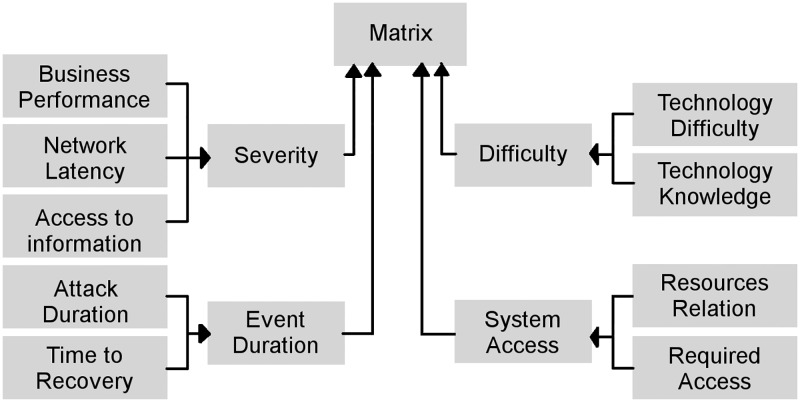
Indexing values. Different indices and values used to create the Matrix of Experts. The different inputs are weighed applying the corresponding correction values.

**Fig 3:**
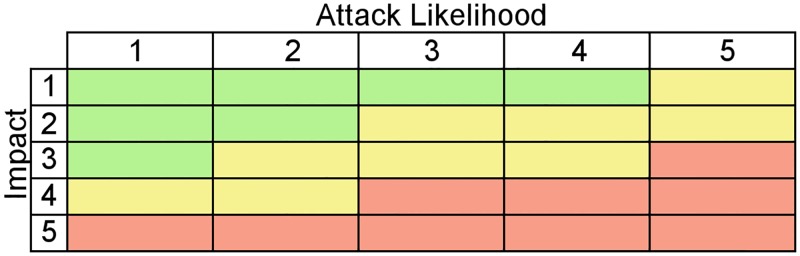
Risk model distribution. The matrix shows the three areas of concern: Red denotes high and medium-high risk; Yellow indicates medium risk and borderline values; Green denotes medium-low and low risk.

In security analysis, a Risk Matrix can evaluate diverse security threats of a specific system based on different parameters as the severity, difficulty, and duration of the event and the relative access to information, not only from the attacker but also from system users. This information identifies the real impact of an attack on the system and the likelihood of this attack’s occurrence, which determine the attack’s risk level. As shown in Figs 1 and 2, system status, infrastructure information, attack information, and risk characteristics were used in order to form a matrix, which would be ranked as is shown in Fig 3.

### Indices and values

The first step was to generate the Matrix of Experts. The values used in the matrix, as shown in Table 1, were provided by a small group of experts in network security with ample experience in network auditing and risk assessment. The Matrix of Experts [24] used in this work had nine indices: Business Performance, Network Latency, Access to Information, Attack Duration, Time To Recover, Technical Difficulty, Technical Knowledge, Resources Relation, and Required Access to System. Each index was evaluated from one to five, being one the lowest and five the highest risk value.

**Table 1:** Attack values general table.

	Values								
*Item*	*BP*	*NL*	*IA*	*AD*	*TTR*	*TD*	*TK*	*ReR*	*RA*
*Level*	1–5	1–5	1–5	1–5	1–5	1–5	1–5	1–5	1–5

Used variables: Business Performance (*BP*), Network Latency (*NL*), Access to Information (*IA*), Attack Duration (*AD*), Time to Recover (*TTR*), Technical Difficulty (*TD*), Technical Knowledge (*TK*), Resources Relation (*ReR*), and Required Access (*RA*). All variables have a minimum of 1 and a maximum of 5.

All the variables used to construct the Risk Matrix contribute to four main inputs as described before. These four main inputs for the Impact and Likelihood values are: Severity, Even Duration, Difficulty, and System Access.

#### Severity

The severity of an attacks represents how stiff the effects of such attack are to the system. In our case the severity of an attacks depended on three values: Business Performance, Network Latency, and Access to Information. Those values were defined as follows:

The **Business Performance** variable was defined as the effect that an attack had to the expected business operations. Similar metrics can be found in other works such as in [28]. This index was related to how much the attack affected the system from a business point of view. A high value of the business performance index (*BP* ≥ 4) meant that the attack affects the business performance, up to the point of stopping normal operations (*BP* = 5). A low index value (*BP* ≤ 2) denoted that an attack had little or no effect (*BP* = 1) in regular business operations. Since VLC is defined at the physical (PHY) and data-link (MAC) layers of communication [1], the difference between the use of this technology and other technologies at the physical and data-link layers does not increase nor decrease the effect on the business performance. However, the use of this variable was required to properly evaluate the risk that the attacks studied posed to the system.

For this work, the **Network Latency** variable was defined as the observed incremented on the time system response when attacked. Metrics such as the ones used in [29] could be applied for greater detail. However, in order to simplify and clarify the analysis, the metric was used as stated applying a more general definition as the one used by [30]. A high (*NL* ≥ 4) latency value represented that a large increment of time was required to get any information through the network due to the network state. On the opposite side, a low latency value (*NL* ≤ 2) represented that the attack had no observable effect over the time required for the information to access and traverse the network. Since VLC data speed ranges from 11.64 Kb/s to 96 Mb/s [1], with higher bit-rates, the channel is generally more susceptible to noise, and an increase of noise requires re-transmissions among other delaying processes. In the cases of lower data rates, attacks over the VLC channels may have no significant difference from those done in any other technology. However, when considering attacks over a VLC channel that is configured for higher transmission data rates, the effects on the latency of the system may become more important and a higher value should be used in this variable. This consideration should be taken into account when using even higher data rates as proposed in [31].

The **Access to Information** index represented the quality and quantity of information that was accessed by the attacker while performing the attack as defined in [32]. A high value of the access to information (*IA* ≥ 5) meant that the attacker had access to a large range of information, including confidential information such as accounting or sales practices. A small value of the access to information index (*IA* ≤ 2) meant that the attacker had access to a small range of information with little intrinsic value such as public information or the network’s name. As in other technologies, the access to information value depends on the attack and not on the implemented VLC system. Still, this parameter was required to properly gauge risk.

#### Duration of the Event

The Duration of the Event represented the total time the attack had an effect over the system from the attacks starts until the full system recovery. It depended on two values: Attack Duration and Time to Recover. For this work, those values were defined as follows:

The **Attack Duration** index represented the time length that an attack was considered active. A high value of duration (*AD* ≥ 4) represented an attack that was active for extended times such as hours or days. The worse case (*AD* = 5) was that the attack has permanent effects over the system. A small value (*AD* ≤ 2) of the duration index represented attacks that were active for short periods of time or were near instant (*AD* = 1) in they duration or effects over the system. Duration as was defined in this work, together with Time to Recover, can be found in other works, such as [33], to measure the impact that an attack would have in a network. As in other technologies, the duration value depends on the attack and not on the implemented VLC system.

The **Time To Recover** index represented the length of time required for the network to recover its normal functions and responds after the end of the attack [32]. A high value of the index (*TTR* ≥ 4) represented that the time required to recover was large, hours or days for example. The worse case scenario (*TTR* = 5) would be that the effects of the attack were, or were near, unrecoverable after the end of the attack. A small value (*TTR* ≤ 2) of the index represented that the networks recovered from the attack effects after a short time or even instantly (*TTR* = 1) after the attack ended.

In the case of VLC versus other systems such as WiFi, the recovery protocols used in VLC have been less tried, deployed and tested, with the exception of the ones ported from other communication technologies. A decrease in performance from such protocols in VLC could be expected, and further testing is required. If the recovery time increased, a higher index of this variable should be considered. Additionally, due to network design, flooding attacks may affect more than a single VLC access point. This flooding, in turn, may trigger a cascading effect. A cascading effect event would increase the *TTR*, and therefore a higher *TTR* index value should be considered. However, if self healing mechanisms as the ones described at [34], the *TTR* value could be expected to decrease.

#### Difficulty

The Difficulty represented the global attack difficulty from the point of implementation to the point of interpreting the attack results. As such, it value came from the combination of the Technical Difficulty and the Technical knowledge required to implement and understand the results of such attack. For this work, those values were defined as follows:

The **Technical Difficulty** index represented how laborious was to implement the technological means of the attack as laid by [35]. A high value of the index (*TD* ≥ 4) meant that implementing the attack was not only feasible and without difficulties, but trivial (*TD* = 5). A low value of the index (*TD* ≤ 2) represented that the technical difficulty of implementing, such as constructing special equipment, an attack was quite high. The reason to use a high index value for low difficulty and low index value for high difficulty was based on the implementation of the Difficulty value (*ADif*_*x*_) of the attack as it was obtained by direct relation of the indexes values.

One of the variables taken into account when evaluating the technical difficulty of the attacks was the almost lack of literature that deals with VLC exploits. The only research related to exploiting the technology that could be directly applied to VLC was related to the Zigbee (Advanced Encryption Standard Counter with CBC-MAC usually referred as AES-CCM*) cryptography [36–38], also used in VLC [1]. Due to the lack of literature, attacks must be designed from the ground up, which in turn, increases the technical difficulty of complex attacks decreasing the index in this variable. In addition to the lack of attack literature, there is also a lack of commercial solutions. The lack of commercial solutions generates a problem when designing broad attacks since each possible VLC implementation may differ from others and therefore a general attack strategy is hard to implement. For these reasons, the *TD* index for VLC attacks should be decreased if compared to similar attacks on other technologies.

The **Technical Knowledge** index represented the expertise and lore required to implement an attack and interpreted the response of the system to such attack as presented in [35]. A high value of the index (*TK* ≥ 4) represented that the technical knowledge needed to implement and understand the response of the attack was trivial. A low value in the index (*TK* ≤ 2) represented that the technical knowledge required to implement and understand the response of the system to the attack was substantial. The reason for using a high index value for low difficulty and low index value for high difficulty was based on implementation of the Difficulty value (*ADif*_*x*_) of the attack as it was obtained by direct relation of the indexes values.

As in the previous case, the technical difficulty index, there is little to none literature related to VLC security [13–15, 17, 18] which, even when using well-known attacks, increases the knowledge required to perform and evaluate the results of such attacks. Also, as in the previous index, there are no commercial solutions over which test or implement generic attacks. Therefore a lower index value of this variable should be used when we compare the technical knowledge required to implement an attack on VLC vs the one required to implement a similar attack in another well-known technology such as WiFi.

To attack VLC systems is necessary to know of the visible light channel behavior and the influence of the physical and geometrical parameters involved in the data transmission. This requires that the attacker posses ample knowledge of optics and photonics so he can exploit weaknesses in the implementation. This know-how is not usually possessed by the typical attacker which is more familiar with the Radio Frequency (RF) domain used in technologies such as WiFi.

This metric, as well as the Technical Difficulty one, have also been studied in [39] to define the security behavior, an index similar to the ones applied in this work.

#### System Access

The System Access quantitatively represented how easy is to access the system to be attacked. It resulted from combining the Resources Relation and Required to the System values. For this work, those values were defined as follows:

The **Resources Relation** index represented the relation between the resources that the attacker needed to implement an attack, and the resources the victim needed to prevent or mitigate such attack. A high value of the index (*ReR* ≥ 4) meant that the attacker needed considerably fewer resources to implement an attack than the victim to prevent such attack. A low value of the index (*ReR* ≤ 2) represented the cases where the attacker required considerably more resources to implement an attack than the victim to prevent that same attack.

On the one hand, due to the low deployment status of VLC, the hardware and software required to implement an attack must be self-made or adapted. Therefore, the value of this variable should be decreased. On the other hand, depending on the attack characteristics, the hardware needed could be easily accessible and cheap, such as photodiodes, while the defense measures may include up to building redesign. In this latest case, the value would increase. Due to these concerns, VLC may have a higher or lower *ReR* value depending on the attack and the attacks requirements if compared to similar technologies.

The **Required Access to System** index represented the access the attacker needed to the victim’s VLC network to successfully perform the attack. This metric, as well as the Resources Relation one, contributed to the System Access metric. Similar metrics are commonly used, such as in [40] to develop security models that validate the robustness of systems. In the case of Required Access to System, a high index value (*RA* ≥ 4) denoted that little or minimum access, such as from outside the premises, was required to succeed in the evaluated attack. A low index value (*RA* ≤ 2) represented a situation in which the attacker required considerable access, even at data-center level, to pursue successfully the attack.

One of the main limitations of VLC attacks is attenuation of the energy received from a VLC access point (AP) versus the one received from an RF AP. Eq 1 shows the electrical power received ($P_{elec}^{RF}$) from RF where *P*_*Tx*_ is the power emitted, *G*_*Tx*_ is the gain of the emitter, *G*_*Rx*_ is the gain of the receiver, λ is the wavelength and *d* is the distance of the receiver from the emitter. From Eq 1 it can be inferred that the electrical power received inversely decreases to the square of the distance. It can be seen through Eq 2 that the electrical power received from VLC ($P_{elec}^{VLC}$) where *P*_*Tx*_ is the power emitted, *S*(*θ*) is the radiation pattern of the emitter, *G*(*ψ*) is the optical gain, *A*_*eff*_ is the effective area of the receiver, *d* is the distance and *R*(λ) is the receiver responsivity. From Eq 2 it can be inferred that the power received inversely decreases by the distance to the fourth power.

$$\begin{array}{c} \begin{array}{c} P_{elec}^{RF}\propto P_{Tx}G_{Tx}G_{Rx}{\left(\frac{\lambda }{4\pi d}\right)}^{2}\\ P_{elec}^{RF}\propto d^{-2} \end{array} \end{array}$$ (1)

$$\begin{array}{c} \begin{array}{c} P_{elec}^{VLC}\propto {\left[P_{Tx}S\left(\theta \right)G\left(\psi \right)\frac{A_{eff}}{d^{2}}R\left(\lambda \right)\right]}^{2}\\ P_{elec}^{VLC}\propto d^{-4} \end{array} \end{array}$$ (2)

**Fig 4:**
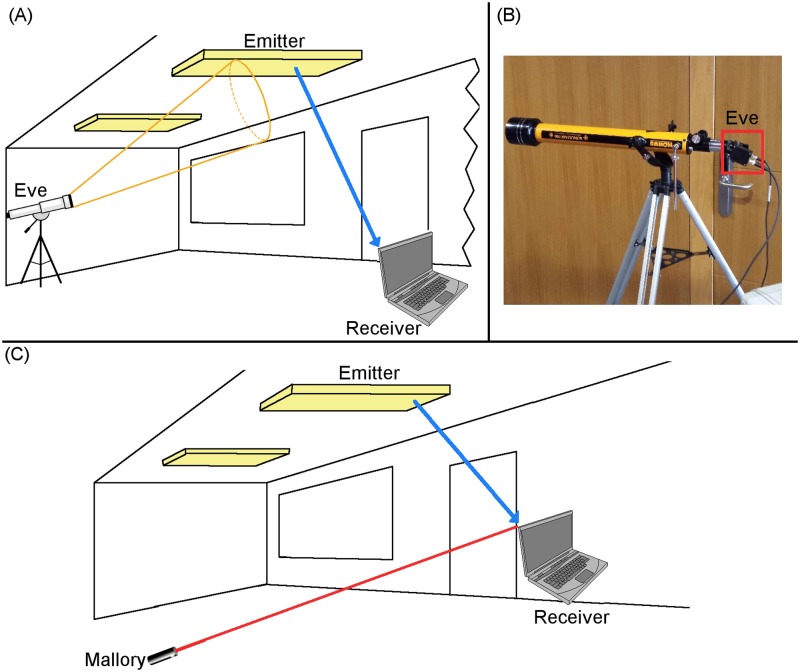
Sniffing and DoS scenarios. (A) Shows an interception of a communication between an emitter (lamp) and a receiver (Computer) by an eavesdropper (Eve) using a photodiode connected to a telescope. (B) Shows the actual assembly of the scenario and the photodiode is highlighted with a red box. The (A) scenario and image (B) were obtained from [18]. (C) Shows a scenario in which an attacker used a Laser to “blind” the receiver (Computer) resulting in a DoS type attack.

As can be observed in Eq 2, the power vs distance relation is inversely proportional (∝) to the fourth power instead of inversely proportional (∝) to the square as is the case for RF emissions (Eq 1). This decrease of system efficiency is based on the optoelectric conversion when transmitting using VLC. The end result is that, in the case of VLC, at the same distance the received energy is less than in the RF case. The main result of the faster loss of power is that, basically, it decreases the range from which an attack can be done compared to regular RF/WiFi attacks. Attack range can be increased by improving the gain of the receiver, as using lenses in the case of the receiver (Fig 4) or focusing the energy on an active emitter (Fig 4). For the purposes of this work, those methods to increase the range have been taken into account in the technical difficulty and therefore included in the *TD* index value.

**Fig 5:**
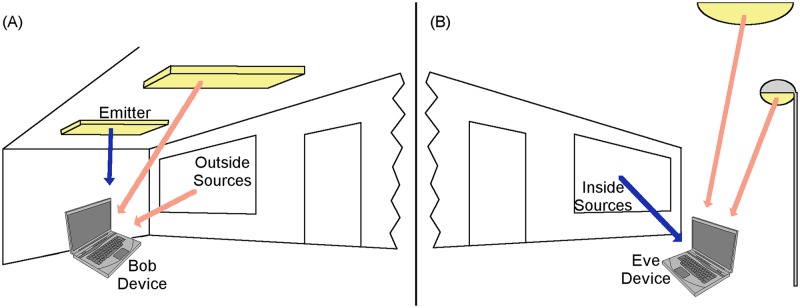
Interference to light channel. Visible light contributions are shown with blue arrows. Noise contributions are shown in red arrows. (A) Indoors scenario where a ceiling light emitting diodes (LED) lamp is the emitter and there are two noise sources: One from the outside through the window and another LED lamp which is not part of the communication with Bob’s device. (B) Outdoors scenario where the communication with Eve’s device comes from the inside through the window (a leak scenario or an indoor-to-outdoor communication scenario) in which two possible noise sources are present: A street lamp and the sun.

Furthermore, the use of multiple emitters as well as the environment, as shown on Fig 5 can increase the noise of the system. This noise limits the signal to noise ratio (SNR) and in consequence, it limits the channel capacity, in other words, it limits the amount of information that can be received through the channel.

The environmental noise, natural or generated, limits the received signal. Applying Eq 3 we can determine the real Signal to Interference and Noise Ratio (SINR). Consequently, multiple emitters, as well as natural sources, limit also the range from which a VLC system may be attacked.

$$\begin{array}{c} SINR=\frac{{\left(P_{Tx}H\left(0\right)R\left(\lambda \right)\right)}^{2}}{\sigma_{Th}^{2}+\sigma_{sh}^{2}+\sum_{i\in I}{\left(P_{i}H_{i}R\left(\lambda \right)\right)}^{2}} \end{array}$$ (3)

On Eq 3, the value *P*_*Tx*_ represents the emitted power, *H*(0) represents the channel gain, *R*(λ) the receiver responsibility, *σ*_*Tx*_ is the thermal noise (based on temperature, bandwidth and amplifier noise figure), *σ*_*sh*_ is the shot noise (based on photo-generated current, the darkness current and background current), *P*_*i*_ is the power of the interfering signals, *H*_*i*_ is the impulse response, *I* is the interfering sources set.

### Determining Risk Matrix values

The global risk level (*NRR*_*x*_) for different attacks over a VLC system was determined by a combination of the Likelihood (*LK*_*x*_) and the event’s Impact (*Impact*_*x*_). The evaluation of Likelihood and Impact is based on the indices enumerated above. Their level descriptions are shown on Table 2.

**Table 2:** Level description of risk.

Level	Risk	Impact	Likelihood
5	Critical	Severe	Probable
4	Serious	Significant	Likely
3	Moderate	Moderate	Possible
2	Minor	Minor	Unlikely
1	Negligible	Negligible	Rare

Relation between the level (numerical) value and the risk, Impact and Likelihood of the studied attack.

#### Determining the impact

As can be observed in Figs 1 and 2 the Impact was obtained from several parameters: Business Performance (*BP*_*x*_), Network Latency (*NL*_*x*_), Information Access (*IA*_*x*_), Attack Duration (*AD*_*x*_), and Time to Recover (*TTR*_*x*_) from the attack. These elements were divided into two categories: Severity of the event (*Sev*_*x*_), and Duration of the event (*T*_*x*_), which were determined by Eqs 4 and 5. On the equations correction factors, as used in [24, 41–43], *α* and *β* were used for severity and duration of the event on Eqs 4 and 5, while the *η* factors were used for the end impact factor as shown in Eq 6.

$$\begin{array}{c} Sev_{x}=\alpha_{1}\left(BP_{x}\right)+\alpha_{2}\left(NL_{x}\right)+\alpha_{3}\left(IA_{x}\right) \end{array}$$ (4)

$$\begin{array}{c} T_{x}=\beta_{1}\left(AD_{x}\right)+\beta_{2}\left(TTR_{x}\right) \end{array}$$ (5)

Network latency had a correlation of 1 for the severity of the event (*α*_2_ = 1.00), while the business performance and access to information had a bigger impact (*α*_1_ = 1.05, *α*_3_ = 1.10) for the severity of the event. In the case of event’s duration, when was considered the attack duration and the time to recover from such attack, it was understood that the attack duration had a larger impact in the event (*β*_1_ = 1.05). Finally, when all variables that affect the attack impact were considered, the severity value had a larger effect (*η*_1_ = 1.10) than the attack duration (*η*_2_ = 1.00). Based on all these considerations the correction values are shown in Table 3.

**Table 3:** Impact correction factors.

	Impact Values						
*Factor*	*α*_1_	*α*_2_	*α*_3_	*β*_1_	*β*_2_	*η*_1_	*η*_2_
*Value*	1.05	1.00	1.10	1.05	0.95	1.10	1.00

This table shows the correction factors. The *α*’s values are used at the severity evaluation. The *β*’s values are used at the duration of the event evaluation. The *η*’s are used at the Impact evaluation.

These elements (*Sev*_*x*_ and *T*_*x*_) contributed to the Impact (*Impact*_*x*_) and were applied using Eq 6. The results were not only numerical but could also be translated into predefined levels. To obtain the values, the correction factors shown on Table 3 were used.

$$\begin{array}{c} Impact_{x}=\eta_{1}\left(Sev_{x}\right)+\eta_{2}\left(T_{x}\right) \end{array}$$ (6)

#### Determining the likelihood

As can be observed in Figs 1 and 2 the Likelihood (*LK*_*x*_) was obtained from multiples parameters: Technology Difficulty (*TD*_*x*_), Technology Knowledge (*TK*_*x*_), Resources Relation (*ReR*_*x*_), and Required Access to the system (*RA*_*x*_). These elements were organized into two sets: Those contributing to the Attack Difficulty (*ADif*_*x*_) value and those contributing to the Access to the System (*AS*_*x*_) values. These values (*ADif*_*x*_ and *AS*_*x*_) were obtained by applying Eqs 7 and 8. The correction factors *γ* and *ϕ* needed for the equations were determined based on the security expertise of the group as in the Impact correction values (*α*, *β* and *η*).

$$\begin{array}{c} ADif_{x}=\gamma_{1}\left(TD_{x}\right)+\gamma_{2}\left(TK_{x}\right) \end{array}$$ (7)

$$\begin{array}{c} AS_{x}=\phi_{1}\left(ReR_{x}\right)+\phi_{2}\left(RA_{x}\right) \end{array}$$ (8)

These elements (*ADif*_*x*_ and *AS*_*x*_) contributed to the Likelihood using Eq 9 and the results were not only numerical but also translated into predefined levels with the corresponding correction (*υ*_*x*_) values.

$$\begin{array}{c} LK_{x}=\upsilon_{1}\left(ADif_{x}\right)+\upsilon_{2}\left(AS_{x}\right) \end{array}$$ (9)

Technology difficulty and technology knowledge contributed equally to the attack difficulty index (*γ*_1_ = *γ*_2_ = 1.00). In the case of the Access to System index, the required access had a larger impact than the resources relation between the attacker and the victim so a small correction factor of 5% was applied. Finally, in the overall likelihood index, the attack difficulty had a larger impact than the access to the system index. For that reason, the small correction factor of 5% was also applied. All correlation factor are shown in Table 4.

**Table 4:** Likelihood correction factors.

	Likelihood Values					
*Factor*	*γ*_1_	*γ*_2_	*ϕ*_1_	*ϕ*_2_	*υ*_1_	*υ*_2_
*Value*	1.00	1.00	1.00	1.05	1.05	1.00

The table shows the correction factors determined by the security expertise of the group. The *γ*’s values are used at the attack difficulty evaluation. The *ϕ*’s values are used at the access to the system evaluation. The *υ*’s are used at the likelihood evaluation.

#### Level descriptions

Five levels were defined for each of the security parameters used in this work. As a general rule, as shown in Table 2, a value of 1 represented the smaller risk and a value of 5 represented the higher risk to the system. Table 2 presents the equivalence of the risk (*NRR*_*x*_), Impact (*Impact*_*x*_) and Likelihood (*LK*_*x*_) values to the numerical value used in Eqs 6 and 9.

The Impact values were obtained from the Severity Value (*Sev*_*x*_ from Eq 4) and the Duration Value (*T*_*x*_ from Eq 5). The corresponding values for the numbers are shown on Table 5.

**Table 5:** Level description of impact values.

Level	Severity	Duration
5	Severe	Perpetual
4	Significant	Long Term
3	Moderate	Moderate
2	Minor	Short Term
1	Negligible	Instant

The table shows the relation between the level (numerical) value and the severity and duration of the event.

The Likelihood values (*LK*_*x*_) were obtained from the Attack Difficulty, *ADif*_*x*_ from Eq 7, and the Access to the System, *AS*_*x*_ from Eq 8, values. The corresponding values for the numbers are shown on Table 6.

**Table 6:** Level description of likelihood values.

Level	Attack Difficulty	Access to System
5	Negligible	Non required
4	Minor	Public
3	Moderate	Controlled
2	Significant	Restricted
1	Severe	Secured

The table shows the relation between the level (numerical) value and the attack difficulty and the required access to the system of the event.

### Risk Rank and risk level

The Risk Rank (*RR*_*x*_) was defined, as shown in Eq 10, by combination of the Impact (*Impact*_*x*_) and the Likelihood (*LK*_*x*_) values.

$$\begin{array}{c} RR_{x}=\varrho_{1}\cdot Impact_{x}\cdot \frac{LK_{x}}{\varrho_{2}} \end{array}$$ (10)

The values for *ϱ*_1_ correction factor were provided by Eq 11 and the values for *ϱ*_2_ correction factor were provided by Eq 12. As can be observed, for all cases, the impact of the attack increases the risk of the event in a more significant way than the likelihood of such event. This was transferred into our equation by applying the *ϱ* values shown in Eqs 11 and 12. These correction factors also helped to identify the attacks that generated higher risk to the system.

$$\begin{array}{c} \varrho_{1}=\left\{\begin{array}{ll} 1.00 & Impact_{x}\leq 3\\ 1.10 & Impact_{x}=4\\ 1.25 & Impact_{x}=5 \end{array}\right\} \end{array}$$ (11)

$$\begin{array}{c} \varrho_{2}=\left\{\begin{array}{ll} 1.00 & LK_{x}\leq 4\\ 0.90 & LK_{x}=5 \end{array}\right\} \end{array}$$ (12)

This value (*RR*_*x*_) was then normalized, a shown in Eq 13, using the summatory of all the possible Risk Ranks ($\sum_{i=1}^{E}RR_{i}$) and the total amount of attacks (*E*) that had been defined for the system.

$$\begin{array}{ccc} NRR_{x}=\frac{RR_{x}\cdot E}{\sum_{i\in E}RR_{i}}; & & x\in E \end{array}$$ (13)

The obtained normalized value (*NRR*_*x*_), also known as risk level, was interpreted to one of the five possible levels used thought this work. The correspondence between the normalized value (*NRR*_*x*_) of the attack and its level is shown in Table 7. The used thresholds were determined in the distribution obtained from the analysis of the considered possible attacks.

**Table 7:** Risk level.

Value	Level
*NRR*_*x*_ > 1.12	High
0.84 < *NRR*_*x*_ ≤ 1.12	Medium-High
0.56 < *NRR*_*x*_ ≤ 0.84	Medium
0.28 < *NRR*_*x*_ ≤ 0.56	Medium-Low
*NRR*_*x*_ ≤ 0.28	Low

The table shows the equivalence between the normalized value (*NRR*_*x*_) of the attack and the level value of the attack. In the normalization, all the attacks were considered in order to generate the corresponding values. The ranges was obtained from dividing the complete range, itself obtained from the difference between the best case (*LK*_*x*_ = 1 and *Impact*_*x*_ = 1) and worse case scenarios (*LK*_*x*_ = 5 and *Impact*_*x*_ = 5), divided in the number of levels considered.

As can be observed in Table 7 the used values were modified by the correction factor (*ϱ*_1_ and *ϱ*_2_). This rate helped to identify the attacks that generated higher risk to the system.

## Example application

As an example of the use of Risk Matrix to determine the quantitative risk of an attack, an analysis was done to four different attacks: War Driving, Queensland alike Denial of Service (DoS), Preshared Key Cracking, and Evil Twin. These attacks were selected to be described in detail since each of them, according to the taxonomy shown in S1 Appendix, is of a different type (reconnaissance, denial, and cracking) of attack and helps understand the methodology used as well as the limitations of VLC technology.

### War driving

The first evaluated attack was the War driving (*WD*_*x*_) one. War Driving is the act of searching for wireless networks by a person in a moving vehicle or walking, using a portable device connected to a network interface in promiscuous mode. The objective of the attack is to detect the existence or not of a data network and its basic configuration parameters. Fig 5 represents a comparable scenario. The index values, normalized average, provided by the groups of experts for the War Driving attacks are shown in Table 8.

**Table 8:** War Driving values.

	Values								
*Item*	*BP*	*NL*	*IA*	*AD*	*TTR*	*TD*	*TK*	*ReR*	*RA*
*Level*	1	1	2	2	1	5	4	4	5

Summary of the variable values for a War Driving attack.

As a reconnaissance attack, the effect of war driving into business performance was low (*BP* = 1). The attack did not increase the latency (*NL* = 1) of the network since it is a passive attack (S1 Appendix). The range of information captured by the war driving attack was low, but it was considered that included information like the use or not of cryptography in the VLC communication as well as the network characteristics. Therefore a medium-low value was given to the Information accessed index (*IA* = 2). The attack duration might be short but it might be repeated thought time so a medium-low value was assigned (*AD* = 1). The required time for the network to recover is usually almost null since, as stated before, war driving is a passive reconnaissance attack (*TTR* = 1). Regarding technical knowledge, easily accessible hardware [18] is required to do this kind of attack so it got a high index (*TD* = 5) value. The information that can be recovered from the attack is also easy to evaluate (*TK* = 4). Since the attacker does not require specialized hardware or many resources and the defender would require physical means to be protected from such an attack, the resources relation was weighted on the attacker side (*ReR* = 5). Finally, since this attack may be done from outside the building or from public areas, the required access index was also high (*RA* = 5). If those emitters could not be observed, then the value should have decreased to zero, making the attack impossible. However, it should be considered the possibility of transmission through transparent elements such as windows.

The value of the attack Impact (2.10) was equivalent a Level 1 (Table 2) Impact value (negligible) due to normalization. The likelihood value (10.25) was equivalent to a level 5 (Table 2) or Probable. These values resulted in a Risk Rank of 5.55. Therefore, the risk level (*NRR*_*WD*_) of the War Driving attack was 0.52, a level 2 equivalent or minor risk attack.

### Queensland alike DoS

The second evaluated attack was the Queensland alike DoS (*QDoS*_*x*_). This attack is a Denial of Service Attack, and therefore a denial phase attack according to the taxonomy used (S1 Appendix). In the Queensland alike DoS, the attacker utilizes a computing device connected to a powerful emitter that makes reception by authorized users impossible due to the interference generated. In the VLC system case, an example of this could be an attacker using a laser, a torch/flashlight or meddling with the existing lamps as illustrated on Fig 4C. The evaluated index values for the Queensland alike DoS attack are shown in Table 9.

**Table 9:** Queensland alike Denial of Service attack.

	Values								
*Item*	*BP*	*NL*	*IA*	*AD*	*TTR*	*TD*	*TK*	*ReR*	*RA*
*Level*	4	5	1	5	2	4	4	4	4

Summary of the variable values for a Queensland alike Denial of Service (DoS) attack.

As a DoS attack [44], the effect of the Queensland alike DoS attack in the business performance is high (*BP* = 4) since it disturbs the access and all translations being done through the VLC system. The attack also significantly increases the latency (*NL* = 5) of the network since it is an active attack and the noise generated decreases the channel capacity. The range of information captured by such attack is negligible, since, by its nature, it only denies access (*IA* = 1). The attack duration can be long and be repeated through time, so a very high value was assigned (*AD* = 5) to the duration. The required time for the network to recover can be expected to be relatively small (*TTR* = 2) and take place moments after the attack stops. In this scenario no self-healing mechanisms were considered since those recovering techniques, as shown in [34], have not been included in the VLC standard [1] or current implementations so the *TTR* value can not be improved. Regarding technical knowledge, the readily available hardware is required to do this kind of attack, so it got a high index value (*TD* = 4). The required knowledge to create and understand the attack is small (*TK* = 4). Since the attacker does not require specialized hardware, as in the case of a flashlight, or many resources, as in a laser, and the defender will require physical means to protect such an attack from outside, the resources relation was weighed on the attacker side (*ReR* = 4). Finally, since this attack may be made from outside the building or public areas, the necessary access index was also high (*RA* = 4).

The value of the attack Impact (7.30) was equivalent a Level 4 (Table 2) Impact value (significant) due to normalization. The likelihood value (8.20) was equivalent to a level 4 (Table 2) or Likely. These values resulted in a Risk Rank of 17.60. Therefore, the risk level (*NRR*_*QDoS*_) of the DoS attack was 1.64, a level 5 equivalent or critical attack.

### Preshared Key Cracking

The third evaluated attack was the Preshared Key Cracking (*PSK*_*x*_) attack. This attack was an exploitation phase attack according to our taxonomy. In this case, the attacker obtains the preshared key from any authorized user or from the existing communication channel. Methods that go from social engineering to code cracking can be used to obtain the key. The evaluated values for the PSK attack are shown in Table 10. Of the listed methods to obtain the key the most difficult one to implement is the one in which the VLC’s cartographic system is broken. VLC uses a variation (CCM*) of the “Counter with CBC-MAC” (CCM) mode for operation on AES [1], as an authentication encryption algorithm. This variation, CCM*, is also used in the ZigBee [45] implementation of the IEEE 802.15.4 standard [46]. CCM* has widely been believed to provide a truly secure method for authentication. However, there have been some demonstrations that prove it is insecure in some specific cases [36–38] that might be exported and exploited in VLC implementations.

**Table 10:** Preshared Key Cracking.

	Values								
*Item*	*BP*	*NL*	*IA*	*AD*	*TTR*	*TD*	*TK*	*ReR*	*RA*
*Level*	3	2	5	5	5	1	1	1	4

Summary of the variable values for a Pre-Shared Key (PSK) cracking attack.

As a cracking/exploitation attack, the effect of the PSK Cracking attack in the business performance had a medium (*BP* = 3) value. This attack may increase the latency slightly due to the extra traffic generated to test the keys (*NL* = 2). The range of information potentially obtainable if the attack success is very high (*IA* = 5). The attack duration can be long and be repeated through time, being limited only if detected by other means or by having the key changed, so a very high value was assigned (*AD* = 5) to the duration index. The required time for the network to recover can be high since once the shared key is obtained the attacker will be able to access the system until the key is changed and all users will need to update their shared knowledge (*TTR* = 5). Regarding technical knowledge, the required hardware will be, or difficult to access or expensive, so the level will be low to do this kind of attack (*TD* = 1). The required knowledge to create and understand the attack is high, so a low value was assigned (*TK* = 1). As noted before, the attacker may require specialized hardware and many resources (*ReR* = 1). Finally, since this attack may be done from outside the building or public areas, the required access index was high (*RA* = 4).

The value of the attack Impact (8.30) was equivalent a Level 4 (Table 2) Impact value (significant) due to normalization. The likelihood value (3.05) was equivalent to a level 1 (Table 2) or rare. These values resulted in a Risk Rank of 4.40. Therefore, the risk level (*NRR*_*PSK*_) of the preshared key attack was 0.41, a level 2 equivalent or minor attack.

### Evil Twin

As an exploitation attack, the effect of the Evil Twin attack (*ET*_*x*_) in the business performance is medium (*BP* = 3). The attack increases the latency but not in high values due to the extra traffic (*NL* = 3). The range of information potentially obtainable if the attack success is very high (*IA* = 5). The attack duration can be long and be repeated thought time so a very high value was assigned (*AD* = 5). The required time for the network to recover can be high since once the twin has been detected the full network needs to be reconfigured or reset (*TTR* = 4). Regarding technical knowledge, the required hardware will be, or difficult to access or expensive, so the level will be low to do this kind of attack (*TD* = 2). The required knowledge to create and understand the attack is high, so a low value was assigned (*TK* = 4). As noted before, the attacker may require specialized hardware such a VLC AP (*ReR* = 3). Finally, even if this attack can be done from outside, generally, the evil twin device would be located inside the premises. Therefore, the required access index would be medium (*RA* = 3). All the evaluated values of the Evil Twin attack are shown in Table 11.

**Table 11:** Evil Twin.

	Values								
*Item*	*BP*	*NL*	*IA*	*AD*	*TTR*	*TD*	*TK*	*ReR*	*RA*
*Level*	3	3	5	5	4	2	4	3	3

Summary of the variable values for a Evil Twin attack.

The value of the attack Impact (9.40) was equivalent a Level 5 (Table 2) Impact value (severe) due to normalization. The likelihood value (6.15) was equivalent to a level 3 (Table 2) or possible. These values resulted in a Risk Rank of 18.75. Therefore, the risk level (*NRR*_*ET*_) of the Evil Twin attack was 1.75, a level 5 equivalent or critical attack.

## Risk classification assessment using a Risk Map

**Fig 6:**
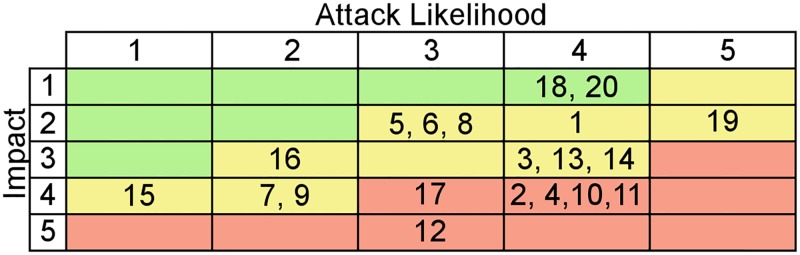
Risk Matrix. The figure shows were each of the considered attacks is located on the Risk Matrix. The values from which the indices are obtained are on the table available at: S1 Table. The attacks are: Beacon Flood (1); De-authentication Flood (2); Authentication Flood (3); Queensland alike DoS (4); Data Reply (5); Frame Injection (6); EAP Downgrade (7); EAP Failure (8); Identity Theft (9); Password Speculation (10); AP Theft (11); Evil Twin (12); MAC Spoofing (13); Man in the Middle (14); PSK Cracking (15); Rogue Access Point (16); Shared Key Guessing (17); Active War Driving (18); Eavesdropping (19); War Driving (20).

The final step of the Risk Matrix approach was the use of a Risk Map for spatial allocation of the attacks. A Risk Matrix that includes all the attacks is shown in Fig 6.

**Fig 7:**
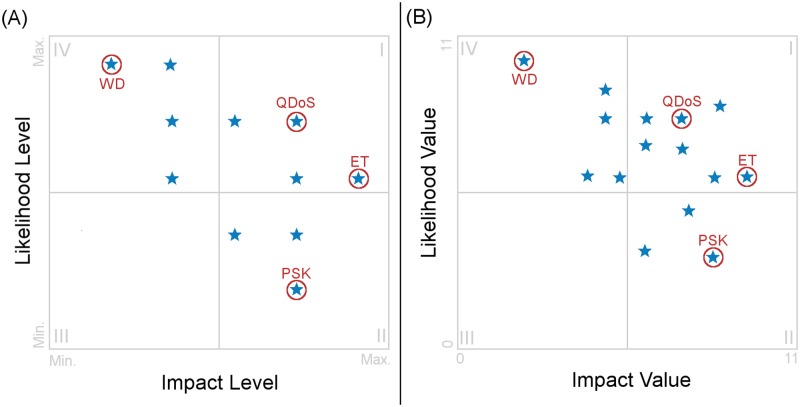
Risk Map. (A) Shows the Risk Map using the Impact and Likelihood levels with normalized values. (B) Shows the Risk Map using the Impact and Likelihood raw values. The different attacks are located in four different quadrants based on their likelihood and impact. Four attacks are circled and identified by their name contractions: War Driving attack (WD); Queensland alike Denial of Service attack (QDoS); Evil Twin attack (ET) and Preshared Key cracking attack (PSK). All the values used are in S1 Table.

As shown in Fig 7 the 20 possible attacks were positioned in four quadrants depending on their Likelihood (*LK*_*x*_) and their attack’s Impact (*Impact*_*x*_) levels. The use of a Risk Map was vital in determining which attacks needed immediate attention and where the efforts to secure a network should be focused. In our case, it helped to determine strategies, that once implemented, decrease the risk improving the security efficiently.

The potentially most dangerous attacks are located within Quadrant I. Therefore special attention should be paid to minimize the risk those attacks presents to our VLC networks. Since the risk is composed by Likelihood (*LK*_*x*_) and Impact (*Impact*_*x*_), in general, and without taking into account the normalization applied, the farther from the central point of the figure the attack is, the riskier the attack will potentially be. In our work, examples of an attack located in this quadrant were the Queensland alike DoS attack and the Evil Twin Attack. Both attacks are tagged with the *QDoS* and *ET* marks on Fig 7.

The attacks with high impact (*Impact*_*x*_) but low likelihood (*LK*_*x*_) are located in the second quadrant. In general, mostly cracking and exploitation attacks will be located here. The reason for their location is due to its difficult implementation, which generates a low likelihood value, and the huge impact they achieve once they are implemented. In our work, an example of an attack located in this quadrant was the Preshared Key Cracking attack. This attack is tagged with the *PSK* mark on Fig 7.

The attacks with low impact (*Impat*_*x*_) and low likelihood (*LK*_*x*_) are located in the third quadrant. Due to their low impact and likelihood, those attacks present a low risk for the network. Therefore, only if other attacks have been deal with, effort should be invested in them. In our work, no attack end up located in this quadrant as can be seen on Fig 7.

Finally, the attacks with a high likelihood (*LK*_*x*_) but low impact (*Impact*_*x*_) are located in the fourth quadrant. In general, reconnaissance attacks will be located on this quadrant. Even if they are easy to implement and use, and therefore have a high likelihood (*LK*_*x*_) they have a, comparatively, low Impact. Nevertheless, attacks located in this quadrant should be addressed, for the reason that, as in the case of reconnaissance attacks, they may be precursors of further and more complex attacks, or so easy to implement that can become a nuisance. In our work, an example of an attack located in this quadrant was the War Driving attack. This attack is tagged with the *WD* tag on Fig 7.

## Discussion

All the indices used during the example application were deduced for a VLC implementation. The physical differences of these networks with other wireless systems, such as WiFi, had been taken into account as well as the media access techniques that they use. Among these considerations were the channel limitations, the medium characteristics, the lack of literature available, the small level of deployment of this technology or the current state of VLC equipment development. All this results in the values obtained through the Risk Matrix methodology as can be shown bellow.

When comparing the values of the four examples, as shown in Table 12, it is observed that the risk of the War Driving and PSK Cracking attacks were almost the same even when they were based in opposite premises: In the case of war driving this was due to its easy implementation which resulted in a high likelihood (*LK*_*WD*_ = 10.25) value but with and low impact (*Impact*_*WD*_ = 2.10). In the case of Preshared Key Cracking attack, the value was due to the attack’s low likelihood (*LK*_*PSK*_ = 3.05) but high Impact (*Impact*_*PSK*_ = 8.30) value. Table 12 list the different *Impact*_*x*_, *LK*_*x*_, *RR*_*x*_ and *NRR*_*x*_ of the evaluated attacks.

**Table 12:** Selected attacks values.

Attack	*Impact*_*x*_	*LK*_*x*_	*RR*_*x*_	*NRR*_*x*_
**War Driving**	2.10	10.25	5.55	0.52
**Queensland**	7.30	8.20	17.60	1.64
**PSK Cracking**	8.30	3.05	5.40	0.41
**Evil Twin**	9.40	6.15	18.75	1.75

The table shows the selected attacks values of: Impact (*Impact*_*x*_), Likelihood (*LK*_*x*_), Risk Rank (*RR*_*x*_) and Normalized Risk Rank (*NRR*_*x*_) of the selected attacks. The full set of values are on the supplied additional material.

As can be observed on Table 12, in the case of the DoS attack, even if it had lower impact (*Impact*_*QDoS*_ = 7.30) than the Preshared Key Cracking (*Impact*_*PSK*_ = 8.30) attack, due to its higher likelihood, (*LK*_*QDoS*_ = 8.20) versus (*LK*_*PSK*_ = 3.05), it got a higher risk (*NRR*_*QDoS*_ = 1.64).

The Evil Twin attack had the almost the same risk (*NRR*_*ET*_ = 1.75) as the DoS attack (*NRR*_*QDoS*_ = 1.64) having a higher impact (*Impact*_*ET*_ = 9.40) but a lower likelihood (*LK*_*ET*_ = 6.15). The same situation happened, but for opposite reasons, when comparing the Queensland alike DoS with the Ware Driving attack: (*Impact*_*QDoS*_ = 7.30) vs (*Impact*_*WD*_ = 2.10) and (*LK*_*QDoS*_ = 8.20) vs (*LK*_*WD*_ = 10.25), resulting in (*NRR*_*QDoS*_ = 1.64) vs (*NRR*_*WD*_ = 0.52). This values ended up converted, using the conversion values of Table 2 in the levels and descriptions of Table 13.

**Table 13:** Examples levels.

Attack	*NRR*_*x*_	Level	Description
**War Driving**	0.52	2	Minor
**Queensland**	1.64	5	Critical
**PSK Cracking**	0.41	2	Minor
**Evil Twin**	1.75	5	Critical

Correspondence of the Normalized Risk Rank (*NRR*_*x*_) with the risk level and its corresponding description.

Once the results were examined, it was apparent that unaccounted risk exists in the networks that employ VLC. According to our study, there were five, out of twenty evaluated attacks, that may present a critical risk for VLC networks. Therefore the implementation of VLC applications will need to add security measures. For example, assuming the existence of elements in the scenarios that leak light, such windows, allows attacks such as War Driving, Queensland alike DoS and Preshared Key Cracking to exist. Minimizing the light leakage, by blocking windows when possible, limits the likelihood of those attacks, and therefore increase the security of the system. However, if there is public access to the premises or the attacker fakes its identity, such in the case of the Evil Twin attack, this kind of protecting measure would have little to no impact in the attack associated risk and further security steps, such as the use of hard encryption, must be taken.

## Conclusion

Even if the Risk Matrix methodology is a valid method to determine risk, and while it is not usually applied to the network security analysis, this works seems to demonstrate that this methodology presents itself as a valid process to determine the quantitative risk of different attacks. Therefore, Risk Matrix and Risk Maps approaches should be considered as a proper starting point in defining the risks that affect a network.

The adequate use of correction factors, based on the researchers and experts experience in the area, defines more accurately the impact and likelihood of the events and attacks studied. Therefore, several consensuated correction factors should be applied to reduce the uncertainty of such analysis while generating a proper distribution of the attacks levels.

By using weighted values, the uncertainty of risk from different attacks is diluted, and as a result, the real impact can be measured and be made more visible. This expected distribution is especially important for the researchers to select the riskier attacks, so the uncertainty of selecting the riskier attacks minimizing the resources is decreased. This optimization of resources is of prime importance in the security arena since, new and more sophisticated attacks appear continuously and may derail the researcher efforts.

The performed risk analysis highlight that even if *a priori* the VLC characteristics on the PHY and MAC layers seem to create a secure medium of communication, VLC implementation and unconsidered elements, such windows, open the possibility of a wide range of attacks that previously has been dismissed, and therefore present substantial risk in VLC use and implementation.

Finally, once all the attacks are positioned on a Risk Map, a clear picture of the relative quantitative risk of the attacks can be observed. This work demonstrated that, from the risk point, the QDoS and the Evil twin attack present the highest risk of all. Moreover, important attention should be paid to attacks such as War Driving since, even if they have a low risk due to their low impact, their likelihood and being the base for other insidious attacks warrant their occurrence.

## Supporting information

S1 Table Attacks weighs. The document includes all the attacks and the weights, factor, and indices used to generate the Risk Matrix according to the methodology utilized in this document. The document also includes statistics values obtained from the indices.
(ODS)

S1 Appendix Attacks taxonomy. The document includes the taxonomy and the attacks classification used in this work.
(PDF)

S2 Appendix List of parameters, symbols and variables. The document includes a full list of parameters, symbols, and variables used in this work as well as a short description of each one of them.
(PDF)

S3 Appendix Glossary of terms. This appendix has been included to help non expert readers in the comprehension of the manuscript. The document includes a list of technical terms used in this work as well as a short description of each one of them.
(PDF)
