# Supplementary material for: Considerations on Visible Light Communication security by applying the Risk Matrix methodology for risk assessment
Source: PLoS One. 2017 Nov 29;12(11):e0188759. doi: 10.1371/journal.pone.0188759 (PMC5706727; doi:10.1371/journal.pone.0188759)
Supplement: S1 Appendix — The document includes the taxonomy and the attacks classification used in this work. (PDF) [file pone.0188759.s002.pdf]

Taxonomy of the Attacks

In order to organize all the attacks for the work presented, several taxonomies can be used, such as in [1]. For the purpose our work, a short taxonomy was used. In it, the attacks were classified by order and by phase. The order classification was based on that type the attack was. The phase classification was based in which part of the sequence of event was the attack implemented.

Classification by Order

As shown in Table 1, attacks were classified as: physical attacks, passive attacks, active attacks, Denial of Service (DoS) attacks and cracking attacks. The classification was not exclusive and a single attack might be classified in more than one division.

Table 1. Classification by Order.

Order	Attack Name
Physical Attacks	Access Point Theft<br>Rogue Access Point
Passive Attacks	MAC Spoffing<br>Eavesdropping<br>War Driving
Active Attacks	Data Reply<br>Frame Injection<br>EAP Downgrade<br>EAP Failure<br>Identity Theft<br>Evil Twin<br>Man-in-the-Middle<br>Active War Driving
DoS/DDoS Attacks	Beacon Flood<br>Authentication Flood<br>De-Authentication Flood<br>Queensland alike DoS
Cracking Attacks	Password Speculation<br>Preshared Key (PSK) Cracking<br>Shared Key Guessing

The table shows in which order (physical, passive, active, Denial of Service and cracking) each of the attacks has been classified into.

Physical attacks

For the purposes of this work, a physical attack was an attack against the physical infrastructure of the VLC network. This VLC infrastructure included, but was not limited to, VLC access points and routers. In the case of the current work, the considered passive attacks were: Access Point (AP) theft and Rogue AP.

Passive Attacks

For the purposes of this work, a passive attack was an attack in which an attempt was made to gain unauthorized information about the network or information being transmitted thought such network, and in which the attacker does not transmit or interact in any way with the network being attacked. In the case of the current work, the considered passive attacks were: MAC spoofing, Eavesdropping and (passive) War Driving

Active Attacks

For the purposes of this work, an active attack was an attack in which an attempt was made to gain unauthorized information about the network or information being transmitted thought such network, and in which the attacker interacted with the network. In the case of the current work, the considered active attacks were: Data Reply attacks, Frame Injection attacks, EAP Downgrade alike attacks, EAP Failure alike attacks, Identity Theft attacks, Evil Twin attacks, Man in the Middle (MiM) attacks and Active War Driving Attacks

Denial of Service Attacks

For the purposes of this work, an Denial of Service attack was an attack in which an attacker tried to limit or deny the access of users to the network. In this work, Distributed Denial of Service Attacks (DDoS) were also considered in this category. In the case of the current work, the considered DoS attacks were: Beacon Flood attacks, Authentication and De-authentication Flood attacks and a Queensland alike DoS attack.

Cracking Attacks

For the purposes of this work, a cracking attack was an attack in which an attacker attempted to break, crack, any of the chryptographic schemes applied to the communication by any mean. In the case of the current work, the considered cracking attacks were: Password Speculation attacks, Pre-shared Key (PSK) cracking attacks and Shared Key Guessing attacks.

Classification by Phase

The attacks were also classified, as shown in Table 2, by the phase (Fig 1) of the intrusion in which the attack is used: Reconnaissance, Denial or Exploitation phase.

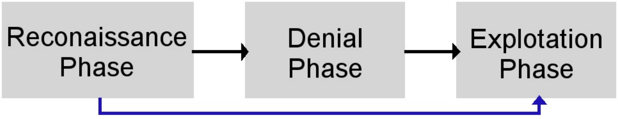

Fig 1. Attacks Classification by Phase. Attacks can be used in the reconnaissance, Denial and Exploitation phases usually following the steps reflected in the figure.

Reconaissance Phase Attacks

For the purposes of this work, a reconnaissance phase attack was an attack in which the attacker investigate, observes and examines the target network in order to find out the network's configuration and if an encryption scheme is used. These types of attacks are usually the first tried, as shown in Fig 1, and provide the attacker with the information required to. latter, exploit possible vulnerabilities and escalate the access to the system.

In the case of the current work, the considered (Table 2) reconnaissance phase attacks were: Passive War Driving attacks, Active War Driving attacks and Eavesdropping Attacks. No Social Engineering attacks were considered for this work even if they are one of the most used reconnaissance attacks.

Denial Phase Attacks

For the purposes of this work, a denial phase attack, not to be confused with a DoS attack, was an attack in which the attacker denies the use of the network to single or multiple users in order to gain access to such a network. These types of attacks are normally used to either cause a break in the network or deny the use of such a network so the attacker may impersonate a user. The attacks occur after a reconnaissance, as shown in Fig 1, and may be an end by itself or be the base of a posterior exploitation phase attack.

In the case of the current work, the considered (Table 2) denial phase attacks were: Beacon Flood attacks, Authentication and De-authentication Flood attacks, a Queensland alike DoS attacks and Mac Spoofing

Table 2. Classification by Phase.

Intrusion Phase	Attack Name
Reconnaissance Attacks	(Passive) War Driving<br>Active War Driving<br>Eavesdropping
Denial Attacks	Beacon Flood<br>Authentication Flood<br>Deauthentication Flood<br>Queensland DoS alike Flood<br>MAC Spoofing
Exploitation Attacks	Data Reply<br>Frame Injection<br>EAP Downgrade<br>EAP Failure<br>Identity Theft<br>Password Speculation<br>AP Theft<br>Evil Twin<br>Man-in-the-Middle<br>PSK Cracking<br>Rogue Access Point<br>Shared Key Guessing

The table list the attacks studied based on which of the three phases the attack was considered to be part of.

Exploitation Phase Attacks

For the purposes of this work, a exploitation phase attack, was an attack in which the attacker exploits vulnerabilities of the system, system implementation or system configuration to gain access to the information transmitted thought the network. These attacks usually take place either after a reconnaissance phase attack or a Denial phase attack as shown in Fig 1. In the case of the current work, the considered (Table 2) exploitation phase attacks were: Data Reply attacks, Frame Injection attacks, EAP downgrade alike attacks, EAP Failure alike attacks, Identity Theft attacks, Password speculation attacks, AP theft, Evil Twin attacks, Main in the Middle (MiM) attacks, PSK cracking attacks, Rogue AP and Share Key Guessing attacks.

References

1. Hansman SL. A Taxonomy of Network and Computer Attack Methodologies. University of Canterbury; 2003. Available from: `http://hdl.handle.net/10092/11201`.
