# Supplementary material for: Considerations on Visible Light Communication security by applying the Risk Matrix methodology for risk assessment
Source: PLoS One. 2017 Nov 29;12(11):e0188759. doi: 10.1371/journal.pone.0188759 (PMC5706727; doi:10.1371/journal.pone.0188759)
Supplement: S2 Appendix — The document includes a full list of parameters, symbols, and variables used in this work as well as a short description of each one of them. (PDF) [file pone.0188759.s003.pdf]

List of Parameter, Symbols and Variables used on the work

"Considerations on Visible Light Communication Security by Applying the Risk Matrix Methodology for Risk Assessment"

Symbol	Description
α_1, α_2 *and* α_3	Correction factors used for the **Severity** variable. Values are shown in Table 3.
β_1 *and* β_2	Correction factors used for the **Duration** variable. Values are shown in Table 3.
η_1 *and* η_2	Correction factors used for the **Impact** variable. Values are shown in Table 3.
γ_1 *and* γ_2	Correction factors used for the **Attack Difficulty** variable. Values are shown in Table 4
ϕ_1 *and* ϕ_2	Correction factors used for the **Access to System** variable. Values are shown in Table 4.
υ_1 *and* υ_2	Correction factors used for the **Likelihood** variable, Values are shown in Table 4.
ϱ_1 *and* ϱ_2	Correction factors used for the **Risk Rank** variable. Values are shown in Equation 11 and 12.
AD_x	**Duration**: Variable used to keep the time length that an attack was considered active.
$ADif_x$	**Attack Difficulty**: Variable used to store the value resulting in combining the TD_x and TK_x variables. The $ADif_x$ variable represents the global attack difficulty from the point of implementation to the point of interpreting the attack results.
AS_x	**Access to the System**: Variable used to keep the value resulting from combining the ReR_x and RA_x variables. The AS_x variable quantitatively represents how easy is to access the system to be attacked.
BP_x	**Business Performance**: Variable used to store the quantitative effect that an attack had to the expected business operation.
DoS	**Denial of Service**: Type of cyber-attack where the perpetrator seeks to make a machine or network resource unavailable to its intended users by temporarily or indefinitely disrupting services.
ET	**Evil Twin**: Type of cyber-attack where a fraudulent access point appears to be legitimate, set up to eavesdrop on wireless communications.
IA_x	**Access to Information**: Variable used to represent the quality and quantity of information that was accessed by the attacker while performing the attack.

Symbol	Description
$Impact_x$	**Impact**: Variable used to keep the criticality and qualitative effect of an attack over the system.
Kb/s	**Kilobits per second**: is a unit of data transfer rate equal to 1,000 bits per second. 125 bytes per second.
LK_x	**Likelihood**: Variable used to keep the plausibility of an attack to happen.
Mb/s	**Megabits per second**: Data transmission rate equal to 1000 Kb/s
NL_x	**Network latency**: Variable used to keep the quantitative observed incremented on the time system response when attacked.
NRR_x	**Normalized Risk Rank**: Variable used to keep the normalized value of RR_x
PSK	**Pre-Shared Key**: Cryptography method in which the keys, or secrets, used to encrypt the communication and share before the communication occurs.
QDoS	**Queensland DoS**: Physical layer DoS attack against Wi-Fi networks. The attack is based on the need of a wireless network to receive the "clear channel assessment"; to determine whether the medium is ready and able to receive data. The attack makes it appear that the medium is busy holding the entire system.
RA_x	**Required Access**: Variable used to represent the access the attacker needed to the victim's VLC network to successfully perform the attack.
ReR_x	**Resources Relation**: Variable used to keep the quantitative value that represents the relation between the resources that the attacker needed to implement an attack, and the resources the victim needed to prevent or mitigate such attack.
RR_x	**Risk Rank**: Variable used to store the value obtained from the relation between $Impact_x$ and LK_x .
Sev_x	**Severity**: Variable used to store the value resulting in combining the BP_x, NL_x and IA_x variables. The Sev_x variable represents how stiff the effects of the attack are to the system.
T_x	**Duration of the event**: Variable used to store the value resulting in combining the AD_x and TTR_x variables. The T_x variable represents the total time the attack has an effect over the system from the attacks starts until the full system recovery.
TD_x	**Technical Difficulty**: Variable used to represent how laborious was to implement the technological means of the attack.
TK_x	**Technical Knowledge**: Variable used to represent the expertise and lore required to implement an attack and interpreted the response of the system to such attack.

Symbol	Description
TTR_x	**Time To Recover**: Variable used to represent the length of time required for the network to recover its normal functions and responds after the end of the attack.
WD	**War Driving**: Type of cyber-attack in which the attacker search for wireless networks in a moving vehicle, using a laptop or smartphone.
