# Supplementary material for: Considerations on Visible Light Communication security by applying the Risk Matrix methodology for risk assessment
Source: PLoS One. 2017 Nov 29;12(11):e0188759. doi: 10.1371/journal.pone.0188759 (PMC5706727; doi:10.1371/journal.pone.0188759)
Supplement: S3 Appendix — This appendix has been included to help non expert readers in the comprehension of the manuscript. The document includes a list of technical terms used in this work as well as a short description of each one of them. (PDF) [file pone.0188759.s004.pdf]

Glossary of terms used on the work "Considerations on Visible Light Communication Security by Applying the Risk Matrix Methodology for Risk Assessment."

Term	Description
5G	5G or 5th generation mobile networks aim for higher capacity than current 4G, allowing a higher density of mobile broadband supporting device-to-device, ultra-reliable, and massive machine communications while having lower latency and battery consumption to the Internet of Things can be implemented.
COTS	Components-off-the-Shelf or Commercially-available-Off-The-Shelf describes components or packaged solutions which are then adapted to satisfy the needs of the purchasing organization, rather than the commissioning of custom made solutions. Motivations for using COTS components include hopes for reduction system whole of life costs since COTS can be obtained at a lower cost over in-house development.
Cracking	Cracking (password) is the process of recovering information from data that have been stored in or transmitted by a computer system. Common approaches include brute-force attacks where the attacker try combinations of characters repeatedly until the password is found.
Cryptography	Cryptography is the practice and study of techniques for secure communication in the presence of third parties by constructing and analyzing protocols that prevent third parties or the public from reading private messages.
DoS	A Denial of Service (DoS) is a computer or network attack where the attacker seeks to make a machine or network resource unavailable to its intended users by temporarily disrupting the services provided by a device. These attacks are usually accomplished by flooding the communication channel or requesting too many resources with superfluous requests to overload systems and prevent some or all legitimate requests from being fulfilled.
Ethernet	Ethernet is a network protocol that controls how data is transmitted over a small size computer network (LAN).
FoV	Field-of-View is the extent of the observable world that is seen at any given moment. In the case of optical instruments or sensors it is a solid angle through which a detector is sensitive to electromagnetic radiation.
GBSB	Geometry-Based-Single-Bounce (model) is a spatial model based on the definition of spatial scattered density function to derive the angle and time of arrival density functions.

Term	Description
Jamming	Jamming is the deliberate blocking or interference with authorized wireless communications, so the content or data transmitted through those channels cannot be received.
LiFi	LiFi or Li-Fi is a bidirectional, high-speed and fully networked wireless communication technology like Wi-Fi. LiFi is a form of visible light communication (VLC) and a subset of optical wireless communications (OWC). LiFi could be a complement to RF communication or even a replacement in contexts of data broadcasting as it is perceived as more secure and it is proposed as a solution to the RF-bandwidth limitations.
SINR	Signal-to-Interference-and-Noise-Ratio is a quantity used to provide theoretical upper bounds on channel capacity in wireless communication systems based on the relation between the signal's power and the result of the interferences power with the noise sources.
Sniffing	Sniffing or Packet sniffing is a technique that allows individuals to capture data as it is being transmitted over a network. This method is used by network professionals to diagnose network problems. It can also be used by malicious users to capture unencrypted data, like passwords and username or to analyze the system behavior to different attacks.
Snooping	Snooping is unauthorized access to another person's or company's data. The practice is similar to eavesdropping and sniffing but is not necessarily limited to gaining access to data during its transmission. Snooping can include casual observance of an e-mail that appears on another's computer screen or watching what someone else is typing. More sophisticated snooping uses software programs to remotely monitor activity on a network.
UWB	Ultra-Wide-Band is a radio technology that can use a very low energy level for short-range, high-bandwidth communications over a large portion of the radio spectrum. It transmits in a manner that does not interfere with conventional narrowband and carrier wave transmission in the same frequency band. UWB is well-suited to short-distance applications, such as PC peripherals and due to its low emission levels tend to be used in short-range indoor applications.
V2V	Vehicle-to-Vehicle communication or Inter vehicle communication is an automobile technology designed to allow vehicles to communicate with each other forming a wireless ad hoc network on the roads. The resulting network created by the V2V members can be used to transmit data relating the vehicle state and therefore increase road safety among other advantages.
